# Analysis of Four Types of Anchorage Devices for Prestressed Glulam Beam and Experimental Research

**DOI:** 10.3390/ma14216494

**Published:** 2021-10-29

**Authors:** Mingfei Li, Mingtao Wu, Nan Guo, Lidan Mei, Yan Zhao

**Affiliations:** 1 College of Civil Engineering, Northeast Forestry University, Harbin 150000, China; a475167970@163.com (M.L.); wmt0830@nefu.edu.cn (M.W.); mld@nefu.edu.cn (L.M.); 2 College of Civil Engineering and Architecture, Wuyi University, Wuyishan, Nanping 354300, China; zhaoyan_hit@163.com

**Keywords:** anchorage device, prestressed Glulam beam, finite element method, stiffness, bearing capacity

## Abstract

An anchorage device is an integral part of the prestressed Glulam beams. Therefore, its rationality and practicability have significant effects on the mechanical performance of the prestressed beams. To investigate the impact of the anchorage devices on the bearing capacity and stiffness of the prestressed beams, this paper compared and analyzed four kinds of anchors in detail through the finite element software. The results showed that when the initial mid-span deflection was 5 mm, 10 mm, and 15 mm, the bearing capacity of prestressed beams with four anchorage devices was 80.37–177.24%, 93.56–182.51%, and 95.62–194.60% higher than that of ordinary Glulam beam, respectively. When the initial mid-span top prestresses were 1 MPa, 1.5 MPa, and 2 MPa, the bearing capacity of prestressed beams with four anchorage devices was 101.71–172.57%, 105.85–175.88%, and 109.64–180.87% higher than that of ordinary Glulam beam, respectively. In addition, based on the simulation results, the prestressed beam with the external anchorage had the highest bearing capacity and stiffness. The deformation capacity of the beam with boot anchorage was the largest. The stress distribution of the beam installed under beam anchorage was the most uniform, and the beam with slotted anchorage was easy to cause stress concentration at the notch. Finally, based on the outstanding performance of the external anchorage, it was selected to carry out one experiment, and the experimental result showed that the simulation could predict the damage model and load–deflection relationship of the prestressed beams well.

## 1. Introduction

As one kind of natural green-building material, timber has many advantages, such as no pollution, low energy consumption, and degradability, which meets requirements for green sustainable development. Therefore, timber-structure buildings get more attention from researchers [1,2,3,4,5,6,7]. Compared with traditional wood materials, the Glulam beam has become an essential part of modern wood structure. It solves the disadvantages of low lumber recovery and complicated processing. It has the advantages of diverse cross-sections, component shapes, fewer centralized defects, higher strength, and more reasonable stress distribution [8,9,10]. However, the Glulam beam often suffers tensile failure due to lower tensile strength, resulting in the compressive strength not being fully utilized. It is a common method to use a variety of reinforced polymers to strengthen Glulam beams. The mechanical properties and failure modes of Glulam beams and basalt fiber reinforced polymer (BFRP) reinforced Glulam beams were compared and analyzed by Chen et al. [11]. Li et al. [12] investigated the influence of different bonding methods of flax fiber reinforced polymer (FFRP) on the flexural performance of Glulam beams. Wang et al. [13] researched the effect of glass fiber reinforced plastic (GFRP) on the bending capacity of Glulam beams. Glišović and Johnsson carried out a series of experiments to study the impact of GFRP on the stiffness, bearing capacity, and ductility of Glulam beams [14,15]. The ultimate bearing capacity of Caribbean pine Glulam beams strengthened with fiber reinforced polymer (FRP) and without FRP were compared and analyzed [16]. Meanwhile, some researchers built the finite element model of near-surface bonded carbon fiber polymer wood beam to study the related influencing factors [17,18,19].

Due to the successful application of prestressing technology in concrete structures, many scholars also began to introduce prestressing technology into wood structures. Some scholars analyzed the flexural ultimate bearing capacity and flexural stiffness of four groups of different linear prestressed reinforced beams [20]. Yang et al. [21] carried out four-point bending tests of carbon fiber reinforced plastic (CFRP) reinforced Glulam beams to make a profound study of the theoretical models of the flexural capacity of unreinforced beams, reinforced beams, and prestressed reinforced beams. Lin et al. [22] demonstrated that the prestress could improve more bending capacity of Glulam beams compared with reinforcement. Zhang et al. [23] concluded that compared with traditional Glulam beams, the prestressed Glulam beams with line and curve steel bars had higher bending capacity. A new type of prestressed laminated beam is proposed, which is prestressed by rotating bolts [24,25,26,27]. Yang et al. found that prestressed beams’ ultimate capacity, stiffness, and ductility were higher than traditional Glulam beams. The prestress loss of prestressed glued wood beam reinforced FRP plate was calculated and analyzed, and then the theoretical analysis was verified by the measured value of reverse arch deflection [28]. Mei [29] proposed a novel prestressed Glulam continuous beam and analyzed the effects of prestressing control on the failure mode, ultimate bearing capacity, and load-deformation relationship of continuous laminated beams. In addition, Mario V. and Alessandra J. [30] dealing with problems inherent to MEMS devices, highlights the procedural process of identifying ghost solutions starting from numerical solutions.

The existing studies have enriched the flexural properties of prestressed material reinforced Glulam beams, but there is a gap in the research on prestressing anchorage devices. Therefore, this paper established finite element models of four different types of anchorage devices (external anchorage device, boot anchorage device, under beam anchorage device, and slotted anchorage device) by using the finite element software ABAQUS. Through the analysis of load-deflection curves and bending stress distribution, the effects of four different types of anchorage devices on the flexural capacity and stiffness of prestressed beams under different prestress and pre-deflection were explored. Finally, based on the finite element analysis results, the four-point bending test selected and studied the anchorage device with the best flexural property. The test measured values were compared and verified with the simulation values.

## 2. Finite Element Experiment

### 2.1. Finite Element Modeling

**Figure 1:**
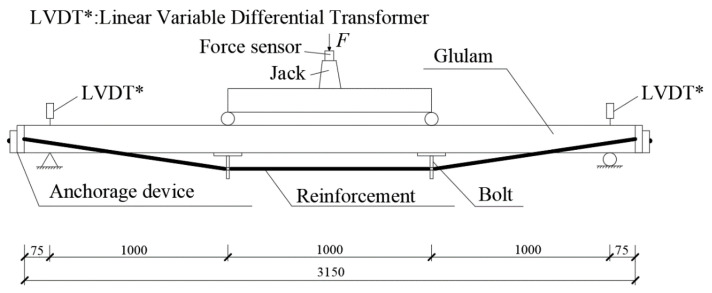
Prestressed beam.

**Figure 2:**
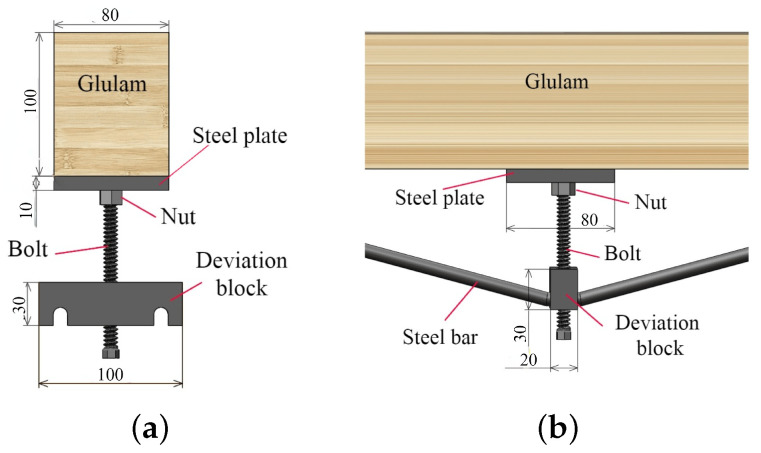
Diagram of screw bolt composition (dimensions are presented in (mm)). (**a**) side view and (**b**) main view.

As shown in Figure 1, the prestressed beam is composed of a Glulam beam, anchorage device, reinforcement, and bolt. Figure 2 presents the diagram of screw bolt composition. All dimensions are in mm, similarly hereinafter.

The Glulam was modeled as anisotropic, and all anchorage devices, screw bolt compositions, and steel bars were modeled as isotropic. First, we built and assembled the parts of the model. Next, the load was applied after the bolt was extended. This simulation included the related prestress and stress state. Subsequently, the bending behavior of prestressed beams was modeled in the quasi-static time-independent domain.

**Figure 3:**
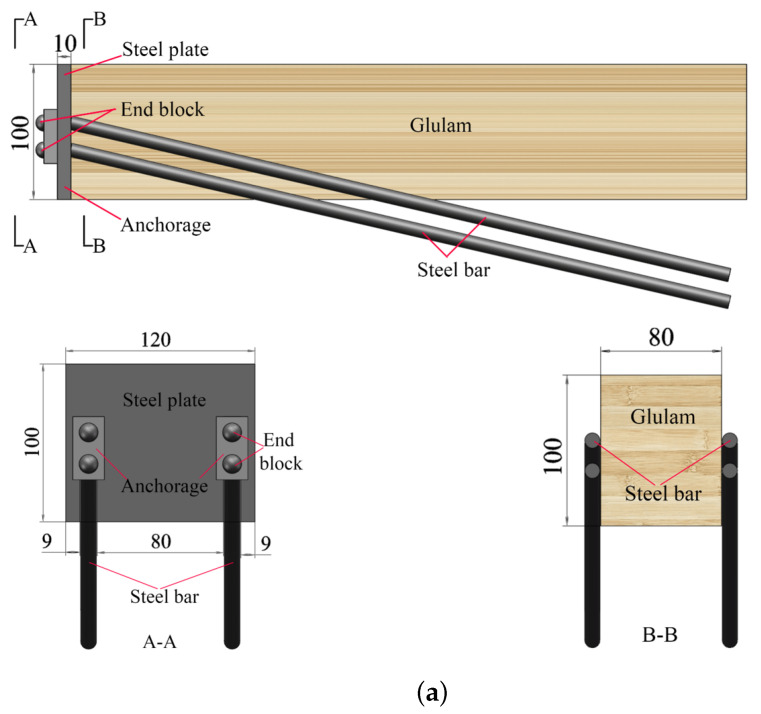

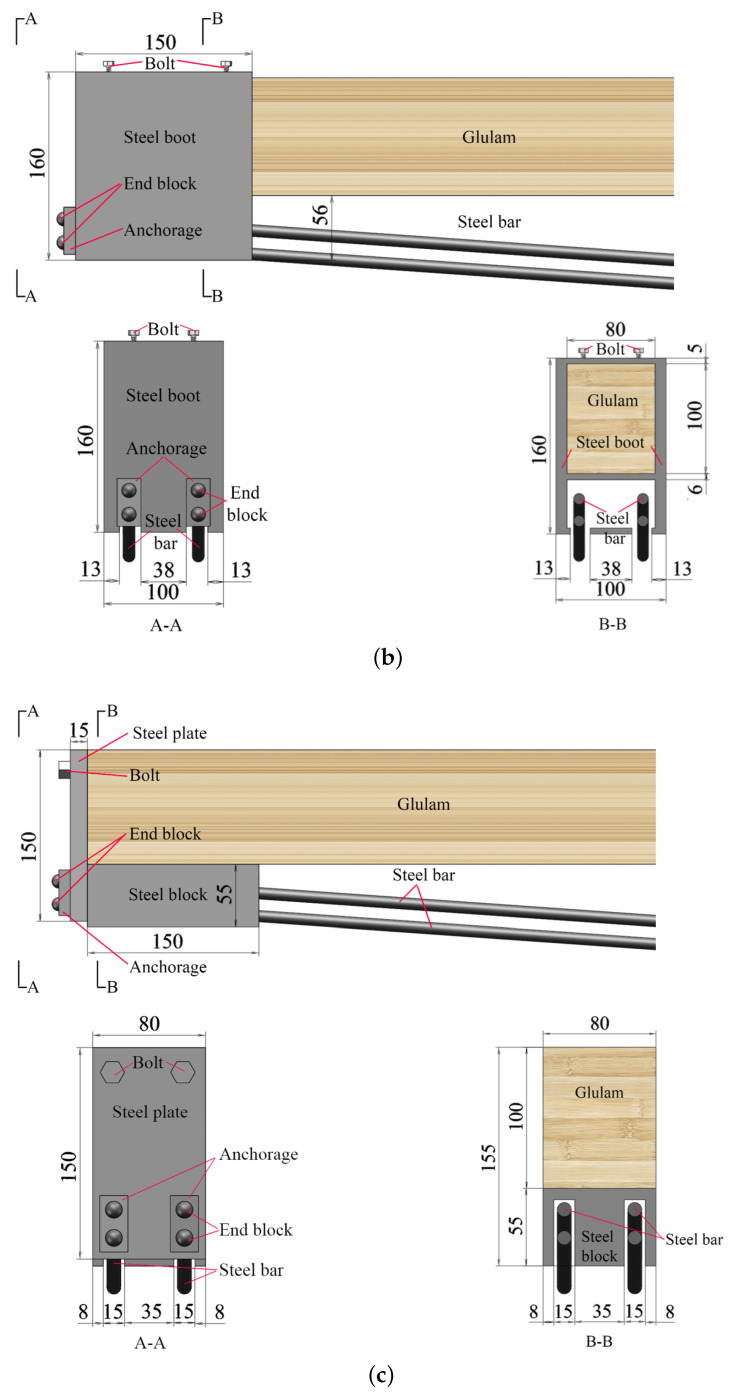

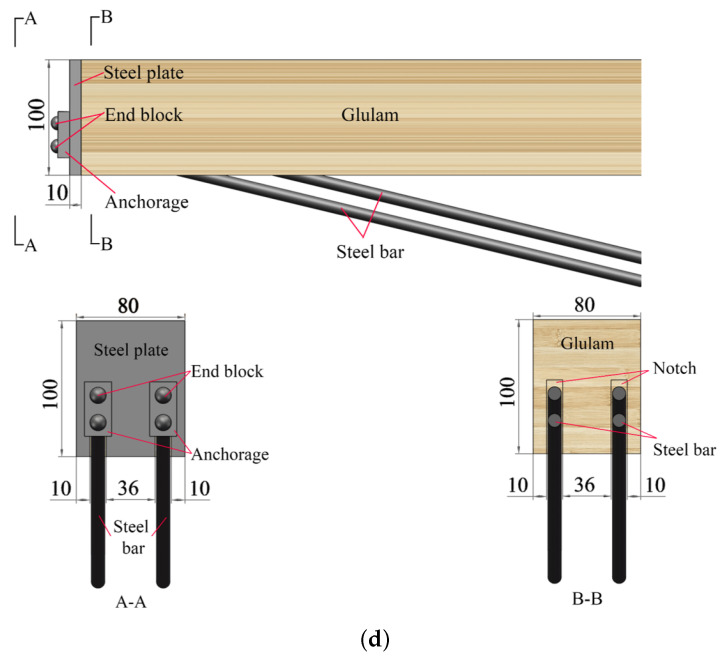
Four types of anchorage devices (dimensions are presented in (mm)). (**a**) mode I: external anchorage device, (**b**) mode II: boot anchorage device, (**c**) mode III: under beam anchorage device and (**d**) mode IV: slotted anchorage device.

There are four types of anchorage devices: external anchorage device, boot anchorage device, under beam anchorage device, and slotted anchorage device, as shown in Figure 3. For convenience, they are named mode I, mode II, mode III and mode IV.

### 2.2. Constitutive Model

The linear orthotropic constitutive equation can be expressed as follows:

(1) $$D=\left[\begin{array}{cccccc} D_{1111} & D_{1122} & D_{1133} & 0 & 0 & 0\\ \; & D_{2222} & D_{2233} & 0 & 0 & 0\\ \; & \; & D_{3333} & 0 & 0 & 0\\ \; & \; & & D_{1212} & 0 & 0\\ \; & sym & \; & & D_{1313} & 0\\ \; & \; & & \; & & D_{2323} \end{array}\right]$$

where

(2) $$D_{1111}=E_{1}\left(1-v_{23}v_{32}\right)r$$

(3) $$D_{2222}=E_{2}\left(1-v_{13}v_{31}\right)r$$

(4) $$D_{3333}=E_{3}\left(1-v_{12}v_{21}\right)r$$

(5) $$D_{1122}=E_{1}\left(v_{21}+v_{31}v_{23}\right)r=E_{2}\left(v_{12}+v_{32}v_{13}\right)r$$

(6) $$D_{1133}=E_{1}\left(v_{31}+v_{21}v_{32}\right)r=E_{3}\left(v_{13}+v_{12}v_{23}\right)r$$

(7) $$D_{2233}=E_{2}\left(v_{32}+v_{12}v_{31}\right)r=E_{3}\left(v_{23}+v_{21}v_{13}\right)r$$

(8) $$D_{1212}=G_{12}$$

(9) $$D_{1313}=G_{13}$$

(10) $$D_{2323}=G_{23}$$

(11) $$r=\frac{1}{1-v_{12}v_{21}-v_{23}v_{32}-v_{31}v_{13}-2v_{21}v_{32}v_{13}}$$

where 1, 2, 3 are, respectively, the longitudinal (*L*), radial (*R*), and tangential directions (*T*) of Glulam (as shown in Figure 4). Therefore, *E*${}_{i}$ is Young’s module in one direction (e.g., *E*${}_{1}$ is Young’s in the longitudinal direction), *G*${}_{ij}$ is the shear module in the certain plane; and *v*${}_{ij}$ is the Poisson’s ratio (*i*, *j* = 1, 2, 3). The cross-section of the Glulam beam is shown in Figure 5.

The above values must satisfy the requirements below:

(12) $$E_{i},G_{ij},v_{ij}>0$$

(13) $$1-v_{12}v_{21}-v_{23}v_{32}-v_{31}v_{13}-2v_{21}v_{32}v_{13}>0$$

### 2.3. Material Properties

**Figure 6:**
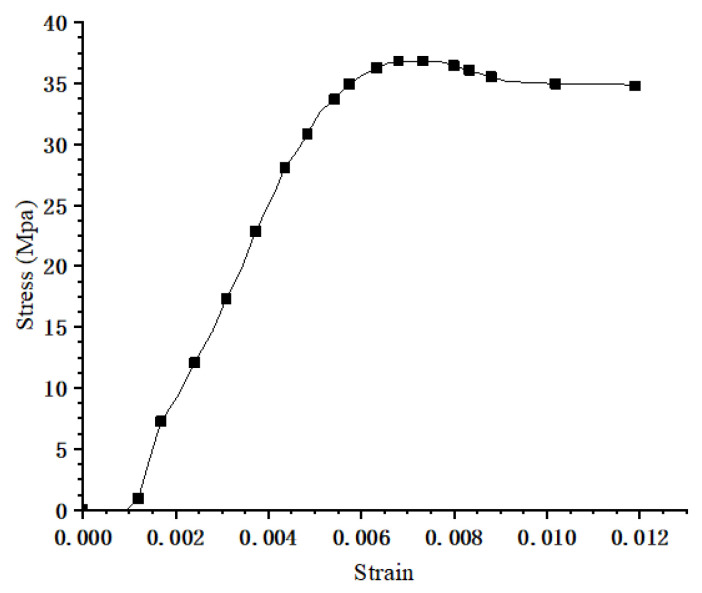
Material compressive properties of Glulam.

**Figure 7:**
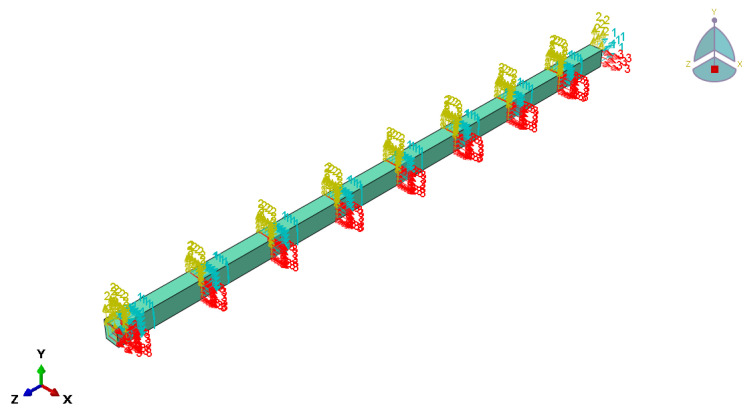
Orientations definition of Glulam.

Material compressive properties of Glulam used in the modeling were from the experimental results, as shown in Figure 6. Based on standards [31,32] and related studies [33], the compressive plastic stress was 35 N/mm${}^{2}$. The orientations definition of Glulam is shown in Figure 7, and the corresponding elastic properties of Glulam are listed in Table 1. The tensile strength and the elastic module of the steel bar were 1720 N/mm${}^{2}$ and 2.0 × 10${}^{5}$ N/mm${}^{2}$, respectively. Furthermore, the radius of the reinforcement was 4.95 mm. On the other hand, the yield strength and the elastic module of other steel members were 345 N/mm${}^{2}$ and 2.0 × 10${}^{5}$ N/mm${}^{2}$, respectively.

### 2.4. Finite Element Simulation

**Figure 8:**
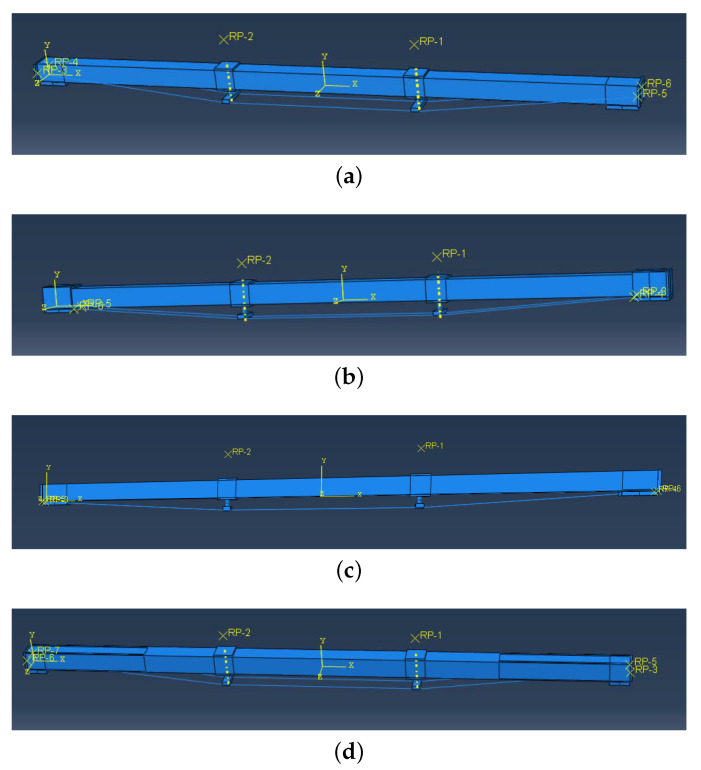
Four prestressed beams models. (**a**) mode I: external anchorage device, (**b**) mode II: boot anchorage device, (**c**) mode III: under beam anchorage device and (**d**) mode IV: slotted anchorage device.

Figure 8 shows the prestressed beams models with four types of various anchorage devices. The surface-to-surface contact was employed to model the interaction between the Glulam and the steel devices, including steel plates and anchorage devices. The former and the latter were defined as the slave surface and master surface, respectively. As there should not be any delamination between anchorage devices and the beams, the laminations were tied. Top prestress and upward pre-deflection were applied to the beams by extending the bolt.

Three-dimensional finite element models were developed to simulate the prestressing state and the loading state after applying the prestress of the prestressed Glulam beams. The Glulam beam was 3150 mm in length, 150 mm in depth, 100 mm in width, and 3000 mm in span length. The mesh size was 20 mm, except for the steel bars, the mesh property of other parts was C3D8R, the steel bars’ mesh property was B31, its size was 50 mm. At the final stage, the flexural tests of prestressed Glulam beams were simulated, following modeling of the prestressing process and then the loading process.

## 3. Results and Discussion

### 3.1. Load-Deflection Curves

The load-deflection curves include two stages, the elastic stage and the plastic stage. At the elastic stage, with load increasing, deflection increases linearly. At the plastic stage, compression plasticity could be better utilized due to the existence of steel bars. Therefore, stages with relatively low slopes could be found in the curves. The upward direction is defined as negative, and the opposite direction is positive.

**Figure 9:**
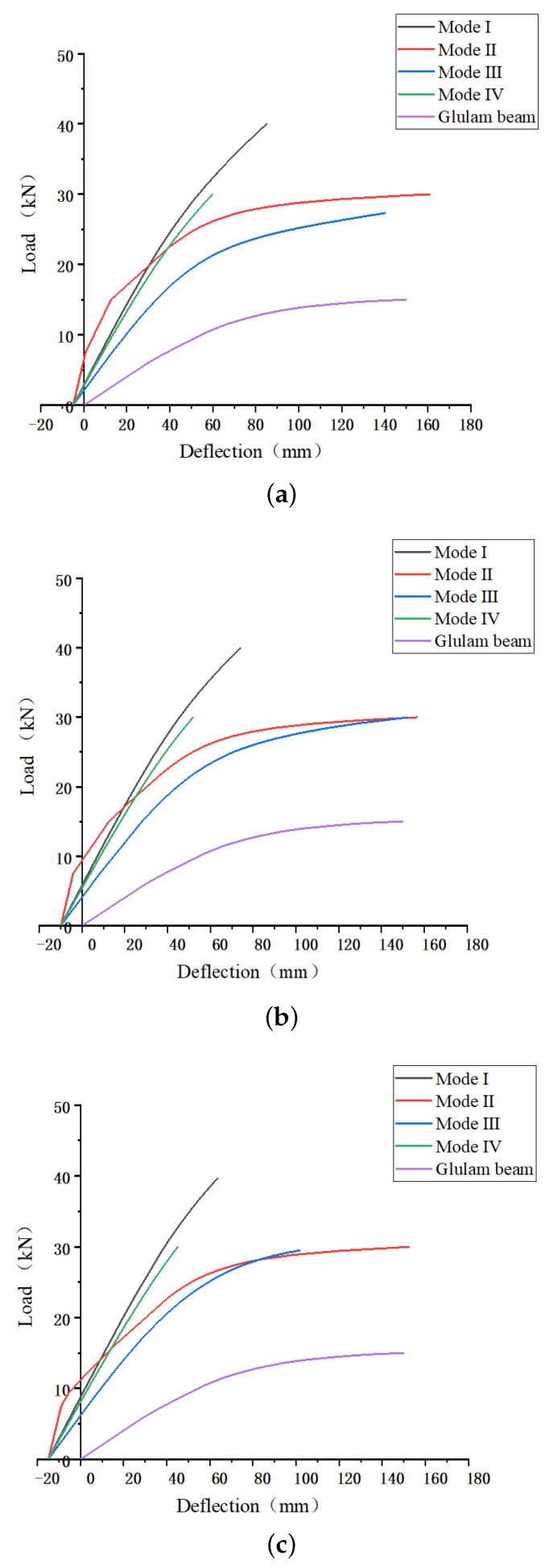
Load-deflection curves with various initial mid-span deflection. (**a**) Five mm initial mid-span deflection, (**b**) 10 mm initial mid-span deflection and (**c**) 15 mm initial mid-span deflection.

Keeping consistent top pre-deflection, control the initial mid-span deflection was 5 mm, 10 mm, and 15 mm, respectively. As is shown in Figure 9, as the initial mid-span deflection increased, the bending capacities of all prestressed beams had certainly improved, and initial deflection is defined as negative deflection. Meanwhile, the stiffness showed significant improvement. Compared with the same size of Glulam beams, the capacity of all prestressed Glulam beams was improved. Within the scope of regulation, the prestressed beams with mode I anchorage device keep the largest bending capacity under the same top deflection. Compared with the other three forms of the prestressed beam, with the initial top deflection increasing, the bending capacity of the prestress beam gets the most significant improvement, but that was still lower than other forms of prestressed beams. At the elastic stage, the stiffness of the beam with mode II anchorage device was the highest, but at a later stage, its stiffness showed a pronounced decline, this kind of beam shows more plasticity. This is because the iron bolt can improve the stiffness initially. Still, with the load increasing, the steel bar could not produce relatively high stress, and the stress concentration could be found in the interface between the iron bolt and beam end.

**Figure 10:**
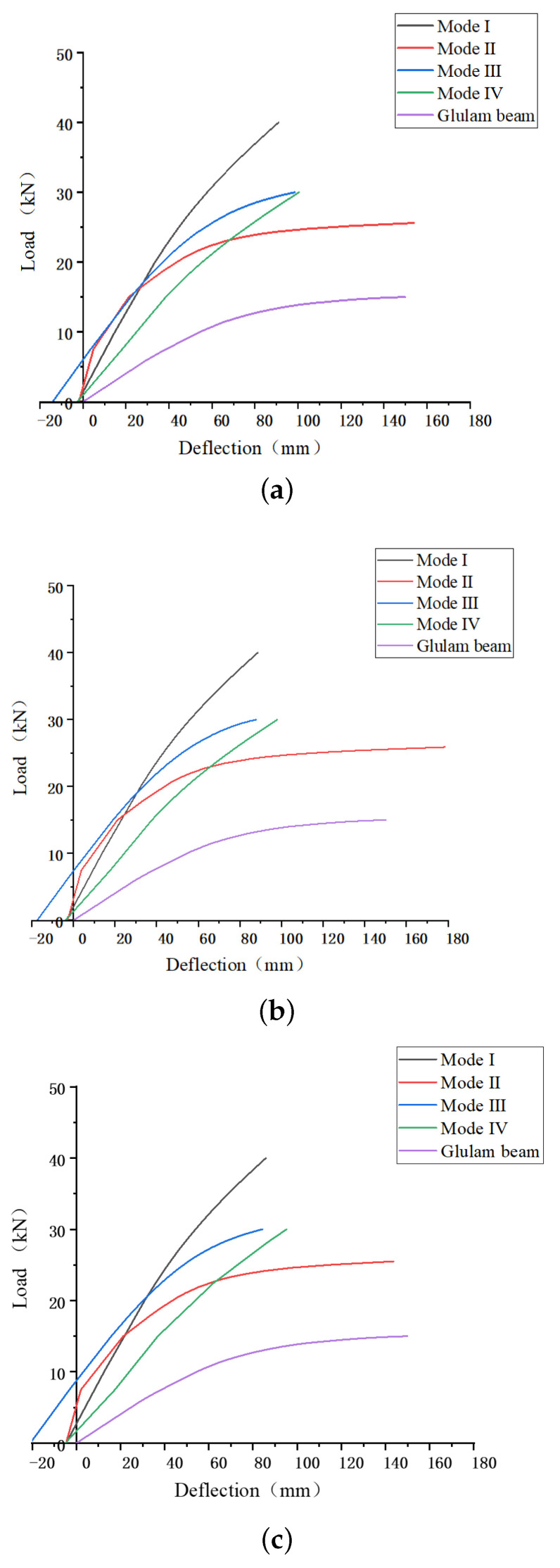
Load-deflection curves with various initial mid-span top prestress. (**a**) One MPa initial mid-span top prestress, (**b**) 1.5 MPa initial mid-span top prestress and (**c**) 2 MPa initial mid-span top prestress.

Next, 1 MPa, 1.5 MPa, and 2 MPa were selected to be initial mid-span top prestress. The load-deflection curves are shown in Figure 10, initial deflection is defined as negative deflection. Compared with the same size Glulam beam without prestressing, the capacity of all prestressed Glulam beams was significantly improved. With the increase of the top mid-span stress, the bending capacity of the prestressed beams does not show noticeable improvement, but their stiffness illustrates certain augment. The bending capacity of the prestressed beam with mode I anchorage device remains the priority, but with the increase of the top prestress, it does not show significant improvement.

On the other hand, the prestressed beam with a mode III anchorage device is easily influenced by the increase of the prestress, and its bending performance and stiffness get the most noticeable improvement. Initially, the stiffness of the prestressed beam with mode IV was the lowest, but when the deformation of the Glulam beam is relatively large, the beam would be in contact with the reinforcement. Therefore, the ability of collaborative work would be improved in the later stage, which will not result in a significant reduction in its stiffness.

### 3.2. The Bending Stress Distribution

**Figure 11:**
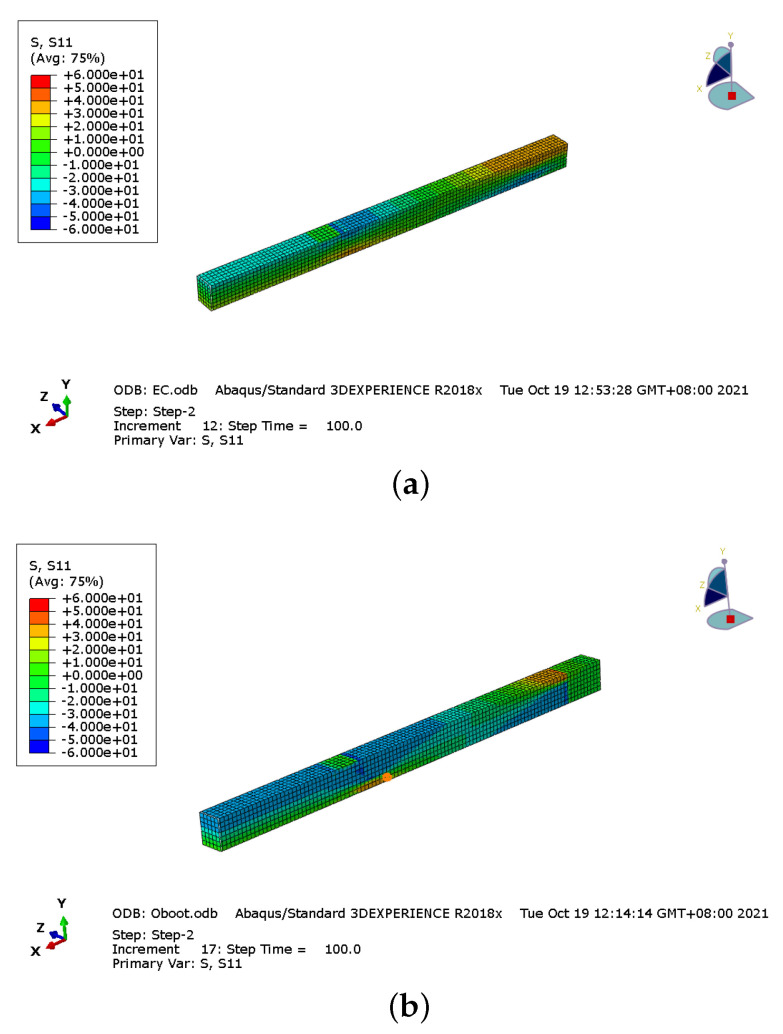

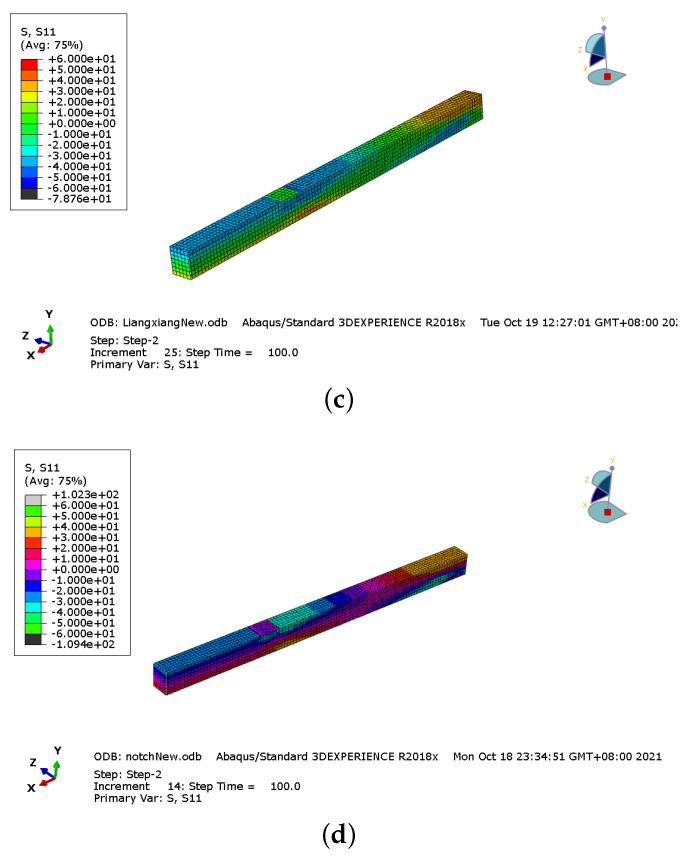
Bending stress distributions of Glulam beams with 30 kN load. (**a**) mode I: external anchorage device, (**b**) mode II: boot anchorage device, (**c**) mode III: under beam anchorage device and (**d**) mode IV: slotted anchorage device.

The bending stress distributions of Glulam beams with 30 kN load are shown in Figure 11. There are the bending stress distribution figures of half-span Glulam beams because of the symmetry. Figure 11a shows the stress distribution of the prestressed beam with mode I anchorage device. Between the two loading points, the stress distribution is uniform. Meanwhile, the stress value is not high, and there is no stress concentration in the whole beam. Figure 11b shows the bending stress distribution of the boot anchorage prestressed beam. The stress distribution between the loading point and midspan is uniform. However, the compression stress between the bending section is higher than in other areas. However, the highest stress is found near the bolt anchorage because of the bolt effect, which causes the stress concentration and stiffness to decline. The compression stress of the prestressed beam with mode III anchorage device between the bending section is uniform, as shown in Figure 11c, but the stress was much high. Figure 11d presents the bending stress distribution of the slotted anchorage prestressed beam. The bending stress near the mid-span is not high. However, as expected, the bending stress near the notch area is very high due to stress concentration caused by the notch.

### 3.3. Impact of Initial Upward Pre-Deflection

When initial deflection was 5 mm, 10 mm, and 15 mm, respectively, the bearing capacity of prestressed beams and Glulam beam is shown in Table 2. Compared with the Glulam beam, the bearing capacity of all prestressed beams is significantly increased by 80.37–194.60%. As the initial deflection increases, bearing capacity is raised to a certain extent. Compared with the prestressed beam with a top deflection of 5 mm, the bearing capacity of the prestressed beam with top deflection of 10 mm and 15 mm is increased by 0–7.31% and 0–8.46%, respectively, and the bearing capacity of mode III is the largest; the stiffness of prestressed beams with top deflection of 10 mm and 15 mm is increased by 1.87–13.30% and 3.46–24.67%, respectively, among which the stiffness of mode IV is the largest. With top deflection increasing, the capacity and stiffness of mode I are always the highest.

### 3.4. Impact of the Initial Top Pre-Stress

When initial top stress was 1 MPa, 1.5 MPa, and 2 MPa, the bearing capacity of prestressed beams and Glulam beam is shown in Table 3. Compared with the Glulam beam, the bearing capacity of prestressed beams was increased by 101.71–180.87%. With the top stress increasing, bearing capacity does not significantly increase, but the stiffness shows noticeable improvement. For example, compared with the prestressed beams with top stress of 1 MPa, the bending performance of prestressed beams with the stress of 1.5 MPa and 2 MPa increased by 0.02–2.05% and 0.49–3.93%, respectively; the stiffness of prestressed beams with the stress of 1.5 MPa and 2 MPa increased 1.19–8.51% and 1.33–11.77%, separately.

## 4. Experimental Research

Based on the FE result and engineering feasibility, the prestressed beam with an external anchorage device (Mode I) could keep better-bending capacity and stiffness. Therefore, it was chosen to do experimental research.

### 4.1. Mechanical Properties of Materials

The raw material was spruce-pine-fir (SPF) from Canada. The dried spruce plates were formed into a rectangular shape with Phenolic adhesives and cured at a temperature of 25 °C. The grade of the timber was I${}_{c}$. Based on standards [31,32] and the former study, compressive and tensile tests were carried out in the laboratory of Northeast Forestry University. The experimental data are shown in Table 4.

All steel specimens were the low-relaxation prestressed steel wire. The nominal yield strength of the steel bar was 1570 MPa, and its tensile strength and elastic modulus were 1640.51 MPa and 1.92 × 10${}^{5}$ MPa, respectively, with a coefficient of variation of 1.71% and 0.35%.

### 4.2. Testing Procedure

The test contained two dimensions of 3150 mm × 80 mm × 100 mm prestressed laminated beams. Specimens fixed with 7 mm diameter low relaxation prestressed steel wires. Specific parameters are shown in Table 5.

### 4.3. Bending Test

**Figure 12:**
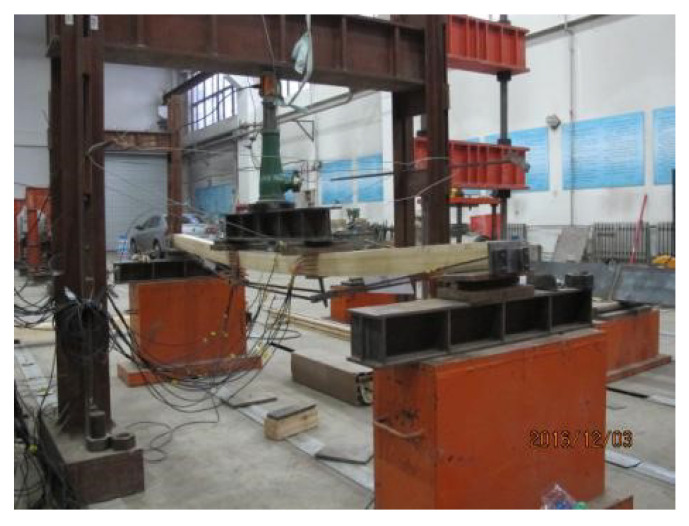
Photo of test.

**Figure 13:**
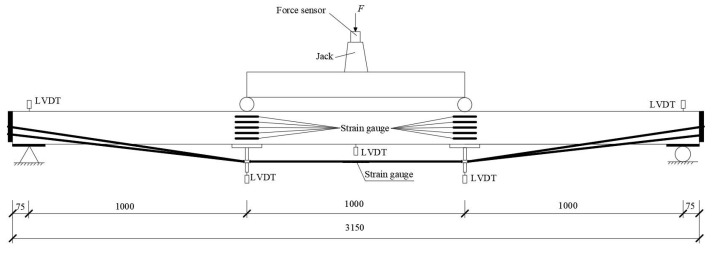
Schematic diagram of the test.

A Four-point bending test was adopted by the Chinese national standards [32,34]. The photo and schematic diagram of the bending test are shown in Figure 12 and Figure 13. Based on a related study [35], the distance between two supports was 3000 mm, and a shear span ( distance from the support reaction force to the nearest applied concentrated load) was 1000 mm. A linear variable displacement transducer (LVDT) with a range of 50 mm was attached to the top side of each specimen at support points to measure the deflections. Two LVDTs and one LVDT with a range of 150 mm were attached to the bottom side of each beam at two loading points and the mid-span point to obtain corresponding vertical deflections, respectively. Five strain gauges were bonded to the loading-point lateral surface with equal spacing to get the strain located at the loading point. Meanwhile, the strain situated at the top and bottom of the half-span beam was gauged by the two 100 mm × 3 mm strain gauges separately. The 2 mm × 3 mm strain gauges were pasted to the steel wire surfaces at an L/2 distance from the beams’ support points.

After assembling the prestressed laminated beam, turning the nut counterclockwise causes the screw to rotate. The steering block moves downward to stretch the reinforcement and simultaneously applies to top prestress and upward pre-deflection to the laminated beams. The prestress value can be controlled by adjusting the distance from the deviation block to the beam bottom.

### 4.4. Failure Modes

**Figure 14:**
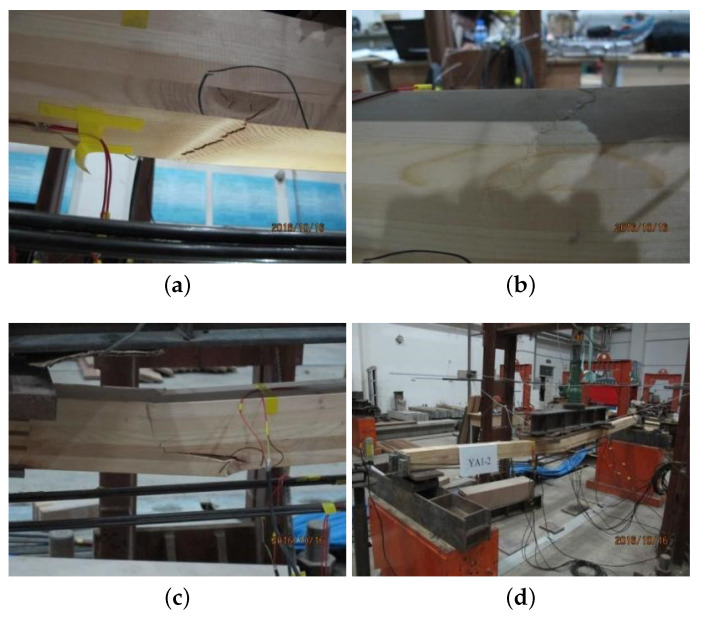
B1 failure mode. (**a**) bottom fibers break, (**b**) top surface wrinkle, (**c**) split above the knot and (**d**) beam failed.

The failure mode of the B1 is shown in Figure 14. The bottom fibers began to break initially when the load was about 85% of the ultimate load due to the knot, as Figure 14a. With load increasing, Figure 14b shows the wrinkle at the top surface near the left loading point of the beam. At this time, the load is 94.61% of the ultimate load. As the load continues to increase, there is a split above the knot, as shown in Figure 14c. Finally, the beam failed due to tension damage at the bottom of the beam, as Figure 14d.

**Figure 15:**
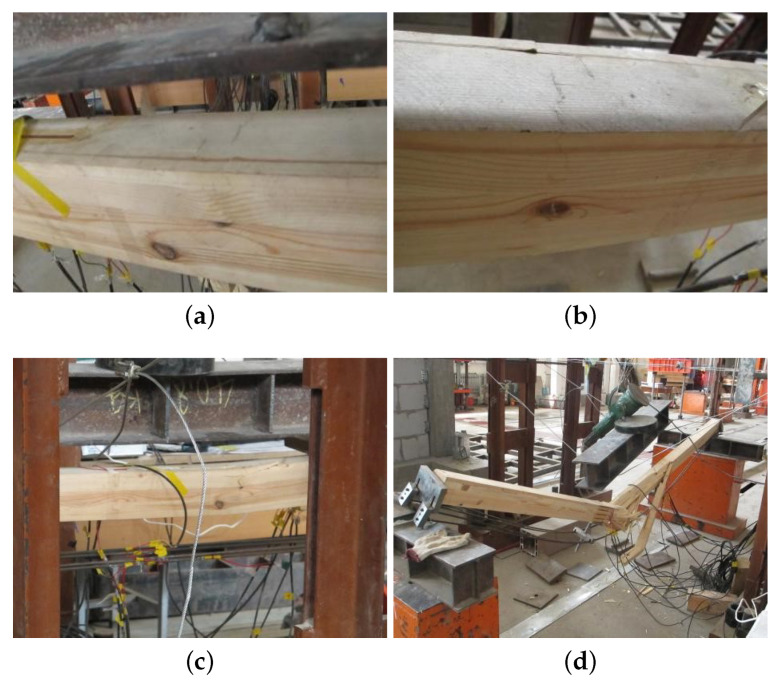
B2 failure mode. (**a**) Top fold, (**b**) fold extends along the section, (**c**) fold extended to the bottom and (**d**) destroyed suddenly.

Figure 15 showed the failure mode of the B2. Initially, there were no apparent changes before the fold shown at the top of the beam, as shown in Figure 15a. As the increase of the load, the fold extends along the section of the beam, as Figure 15b. Figure 15c presented that when the load reached 32 kN, the fold developed to the bottom with reduced bearing capacity. Finally, the beam is suddenly destroyed during the loading stage, as shown in Figure 15d.

**Figure 16:**
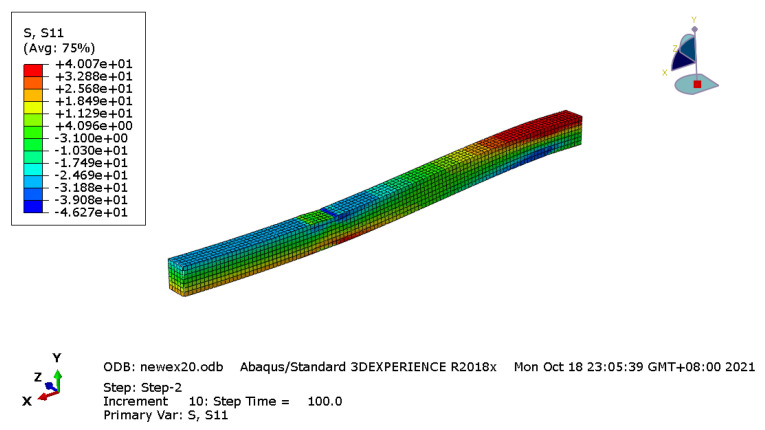
Stress distribution.

This kind of failure mode was consistent with the simulation result. As shown in Figure 16, the tested beam was damaged caused by the top compression damage, the compressive strength of the top area can be fully utilized, and obvious folds could be found in this area.

### 4.5. Load-Deflection Curves and Bearing Capacity

**Figure 17:**
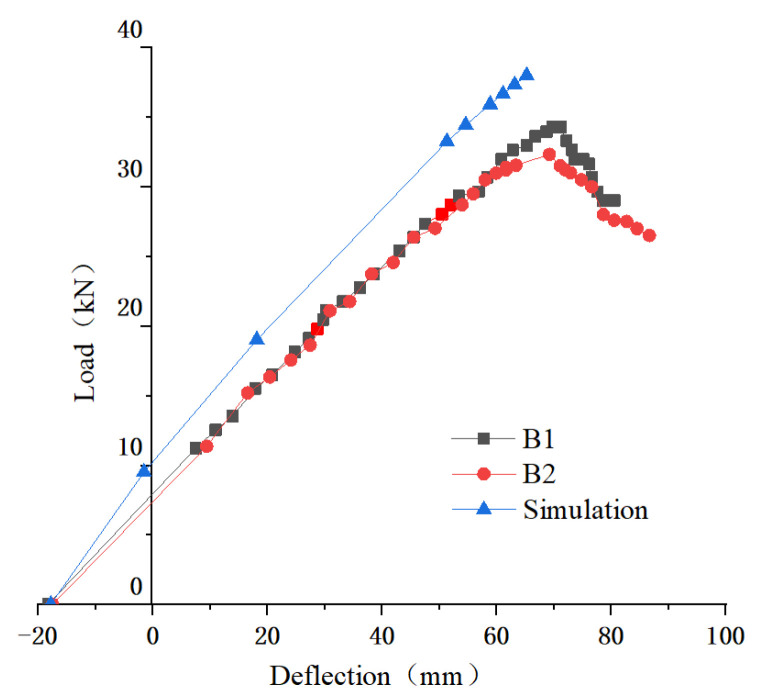
Load–deflection curves of tested beams and simulation.

Figure 17 shows the load-deflection curves of tested beams. Before the peak point, there is no obvious turning point, and the curves are similar to the line, which is identical to the curves of the simulation result. Initial upward deflection is defined as a negative deflection. By comparison, the simulation can predict the load-deflection curves of the tested beams. As the initial defect of the timber was not taken into consideration, the prediction results capacity was higher than the results from the test.

As shown in Table 6, the bearing capacity of the mode I beam is close to the simulation results. However, compared with the simulation results, the stiffness is relatively low due to the difference between initial defects and raw materials of laminated materials.

## 5. Conclusions

- Compared with the Glulam beam, prestressed beams with four types of anchoring devices could be obviously improved. When the prestress is 1 MPa, 1.5 MPa and 2 MPa, the bearing capacity is increased by 101.71–172.57%, 105.85–175.88%, and 109.64–180.87%, respectively; when the top deflection is 5 mm, 10 mm and 15 mm, the bending performance of the prestress beams is improved by 80.37–177.24%, 93.56–182.51%, and 95.62–194.60%;
- Under different prestress and pre-deflection values, the stiffness and bearing capacity of prestressed beam with the mode I is the highest; but the bearing capacity of prestressed beams with the mode III gets the hugest improvement with the initial top stress increasing. Compared with 1 MPa prestressed beams, the bearing capacity of 1.5 MPa and 2 MPa prestressed beams is increased by 8.51% and 11.77%, respectively; compared with the beam with pre-deflection of 5 mm, the bearing capacity of the beam with pre-deflection of 10 mm and 15 mm is increased by 13.30% and 24.67%, respectively;
- Compared with the stress distribution of the other three kinds of prestressed beams, the beam with mode I is most uniform. Therefore the properties of the raw material could be fully utilized;
- Experimental data show the failure mode of the prestressed tested beams is the brittle failure. Compared between experimental results and simulation results, the simulation could better predict the basic linearity of the load-displacement curve, ultimate bending capacity, and the failure mode.

## Figures and Tables

**Figure 4:**
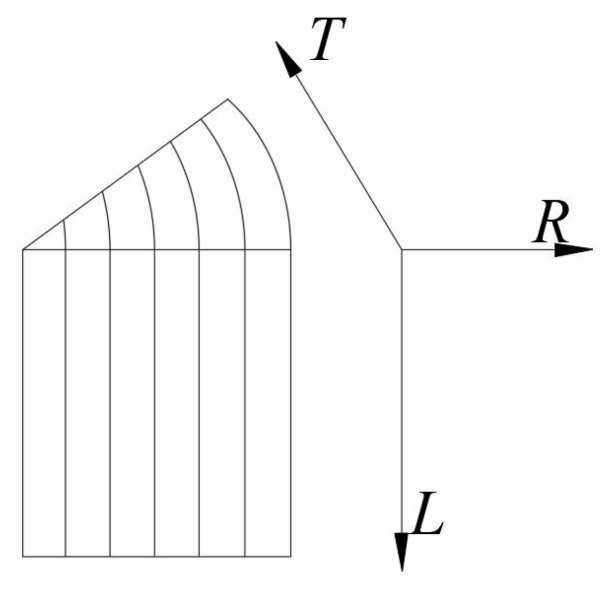
Three-dimensional image of Glulam.

**Figure 5:**
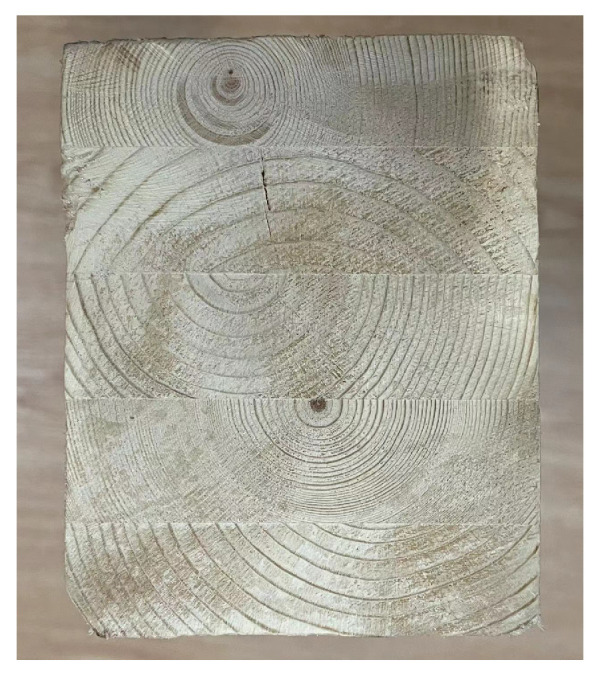
Cross-section of Glulam beam.

**Table 1:** Corresponding elastic properties of Glulam.

D${}_{1111}$	D${}_{2222}$	D${}_{3333}$	D${}_{1212}$	D${}_{1313}$	D${}_{1122}$	D${}_{1133}$	D${}_{2323}$	D${}_{2233}$
8654.084	488.532	488.532	564.574	564.574	221.982	221.982	150.553	182.109

**Table 2:** Bearing capacity with various initial deflection.

Deflection (mm)	Anchoring Device Type	Yield Point Load (kN)	Deflection (mm)	Improvement Rate (%)
-	-	11.34	64.94	-
5	Mode I	31.45	57.38	177.24%
	Mode II	25.31	53.78	123.13%
	Mode III	20.46	55.16	80.37%
	Mode IV	30.00	59.85	164.47%
10	Mode I	32.05	50.60	182.51%
	Mode II	25.20	52.77	122.11%
	Mode III	21.96	52.66	93.56%
	Mode IV	30.00	51.89	164.47%
15	Mode I	33.42	46.70	194.60%
	Mode II	25.30	51.92	123.04%
	Mode III	22.19	46.12	128.55%
	Mode IV	30.00	45.09	164.47%

**Table 3:** Bearing capacity with several top prestress.

Initial Top Pre-Stress (MPa)	Anchoring Device Type	Yield Point Load (kN)	Deflection (mm)	Improvement Rate (%)
-	-	11.34	64.94	-
1	Mode I	30.92	60.46	172.57%
	Mode II	22.88	64.83	101.71%
	Mode III	27.16	69.35	139.43%
	Mode IV	26.97	85.54	137.75%
1.5	Mode I	31.29	59.74	175.88%
	Mode II	23.35	63.18	105.85%
	Mode III	27.18	63.45	139.64%
	Mode IV	26.97	83.42	137.80%
2	Mode I	31.86	56.94	180.87%
	Mode II	23.78	63.97	109.64%
	Mode III	27.42	61.19	141.71%
	Mode IV	27.10	81.55	138.91%

**Table 4:** Experimental data of timber.

Type	Peak Compressive (*f*${}_{\mathrm{cu}}$)	Peak Tensile (*f*${}_{\mathrm{tu}}$)
Strength (MPa)/COV(%)	35/4.63%	81.32/6.42%
Elastic modulus (MPa)/COV(%)	10,350.2/6.14%	10,350.2/6.18%

**Table 5:** Parameters of specimens.

Specimen	Size (mm × mm × mm)	Reinforcement Ratio (%)	Pre-Force (kN)
B1	3150 × 80 × 100	19.24 %	9
B2			

**Table 6:** Data comparison of two beams.

Specimen	A (mm${}^{2}$)	Reinforcement Ratio	Pre-Force (kN)	Bearing Capacity (kN)	Deflection (mm)	Failure Mode
B1	100 × 80	19.24%	9	34.38	69.75	tensile damage
B2				32.33	69.25	compression damage

## Data Availability

The data presented in this study are available on request from the corresponding author.
